# Supplementary material for: New Isolates of Betachloroviruses Shed Light on the Diversity and Biological Complexity of an Unexplored Group of Giant Algal Viruses
Source: Viruses. 2025 Aug 8;17(8):1096. doi: 10.3390/v17081096 (PMC12390592; doi:10.3390/v17081096)
Supplement: Supplementary file 1 [file viruses-17-01096-s001.zip › viruses-3737636-supplementary.pdf]

SUPPLEMENTARY MATERIAL

A) DNA Packaging ATPase

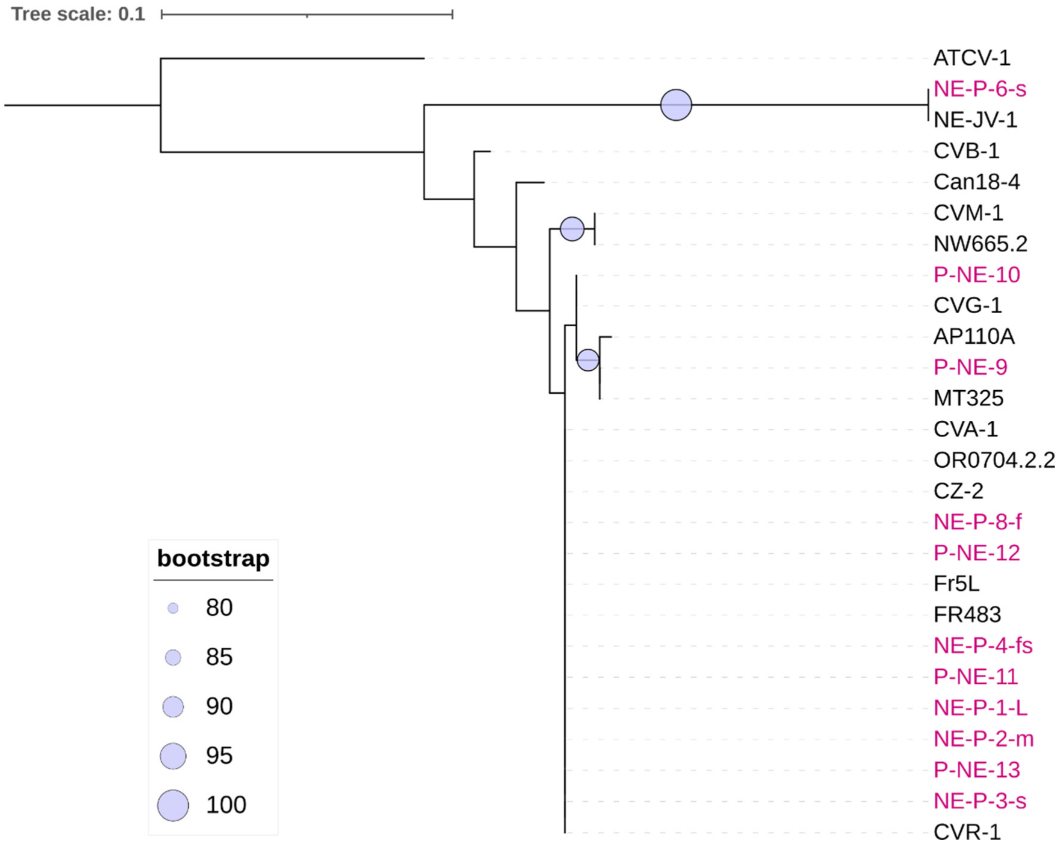

B) DNA polymerase B family

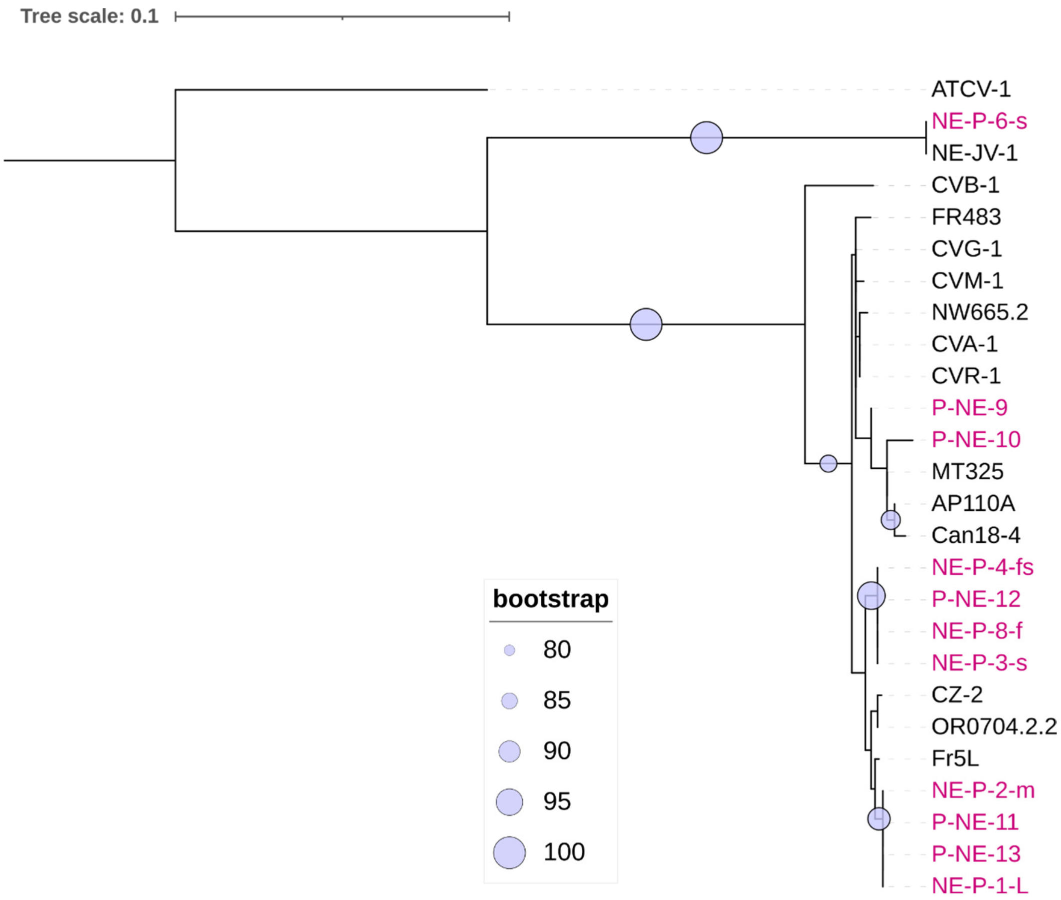

C) DEAD/SNF2-like helicase

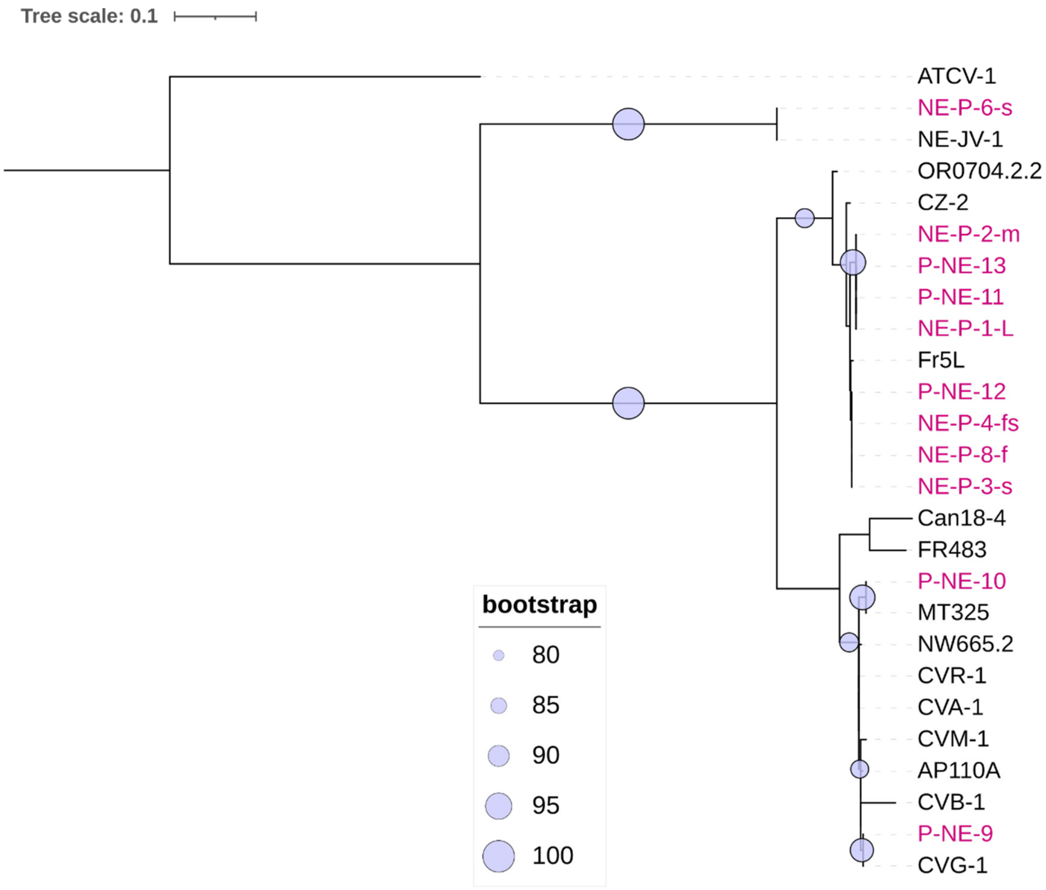

D) Transcription initiation factor IIB

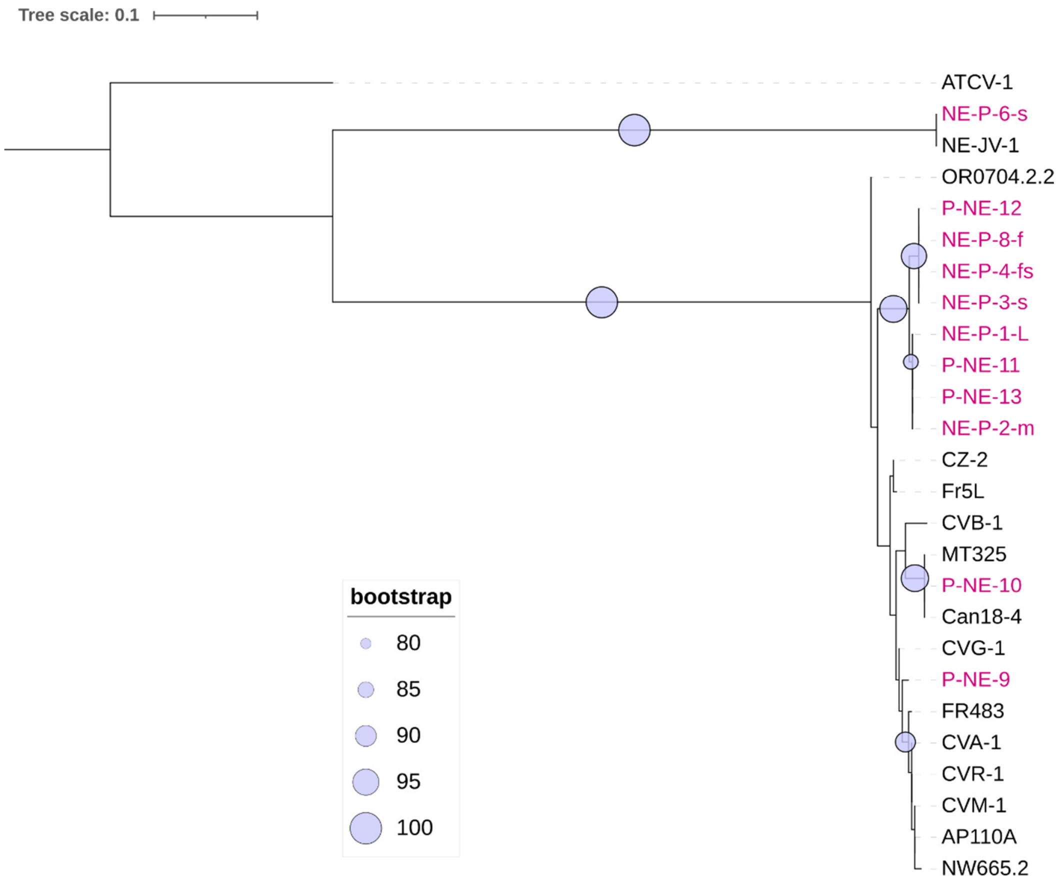

E) DNA topoisomerase II

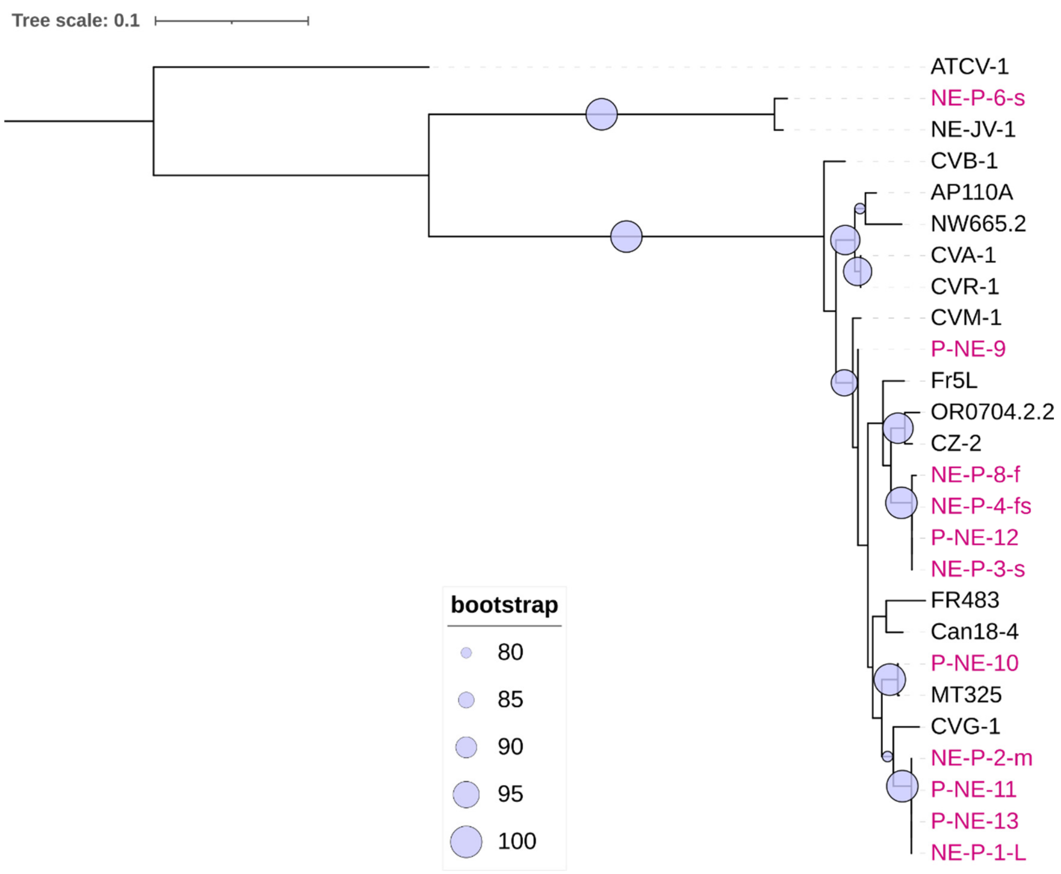

F) Poxvirus Late Transcription Factor 3

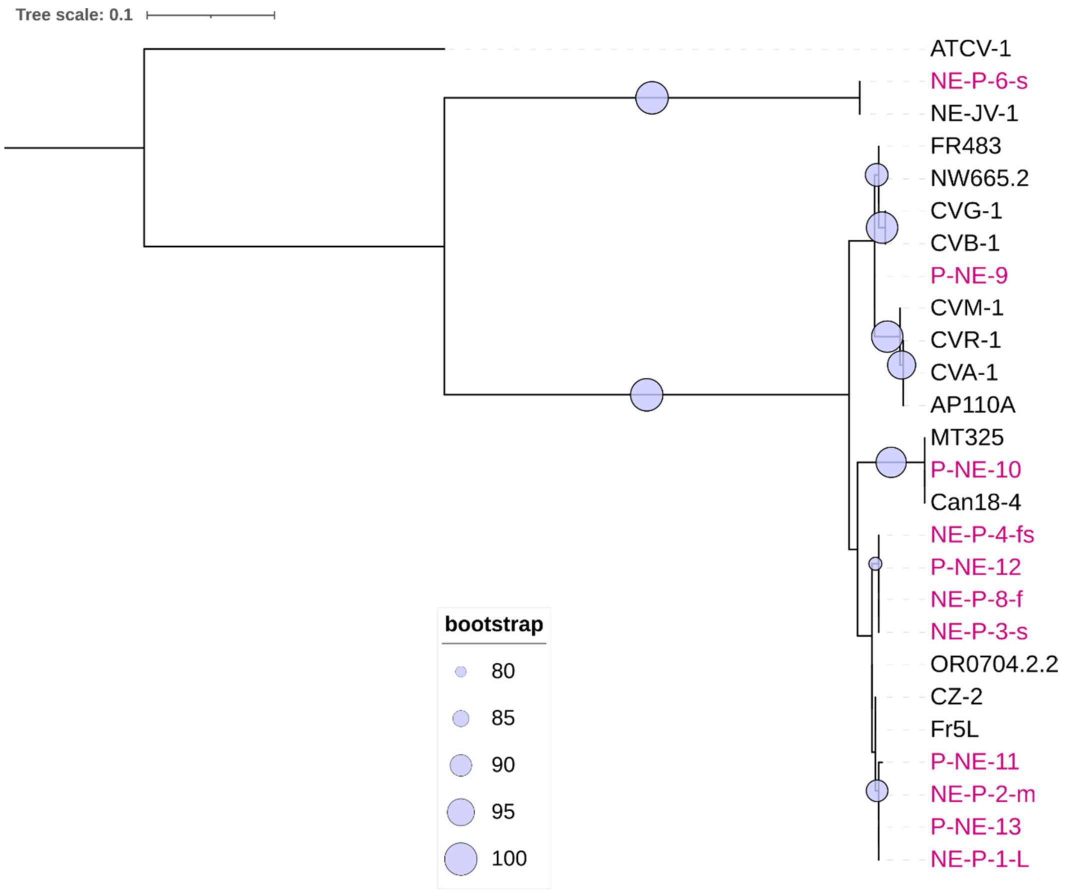

Figure S1: Phylogenetic trees of betachloroviruses. A) A32; B) PolB; C) SFII; D) TFIIB; E) TopoII; F) VLTF3. In all trees, the new isolates are represented in pink labels, while already known isolates are in black. ATCV-1, a gammachlorovirus, was used as an outgroup. Only bootstrap values above 80 are represented. The tree scale refers to the substitution rate of amino acids.

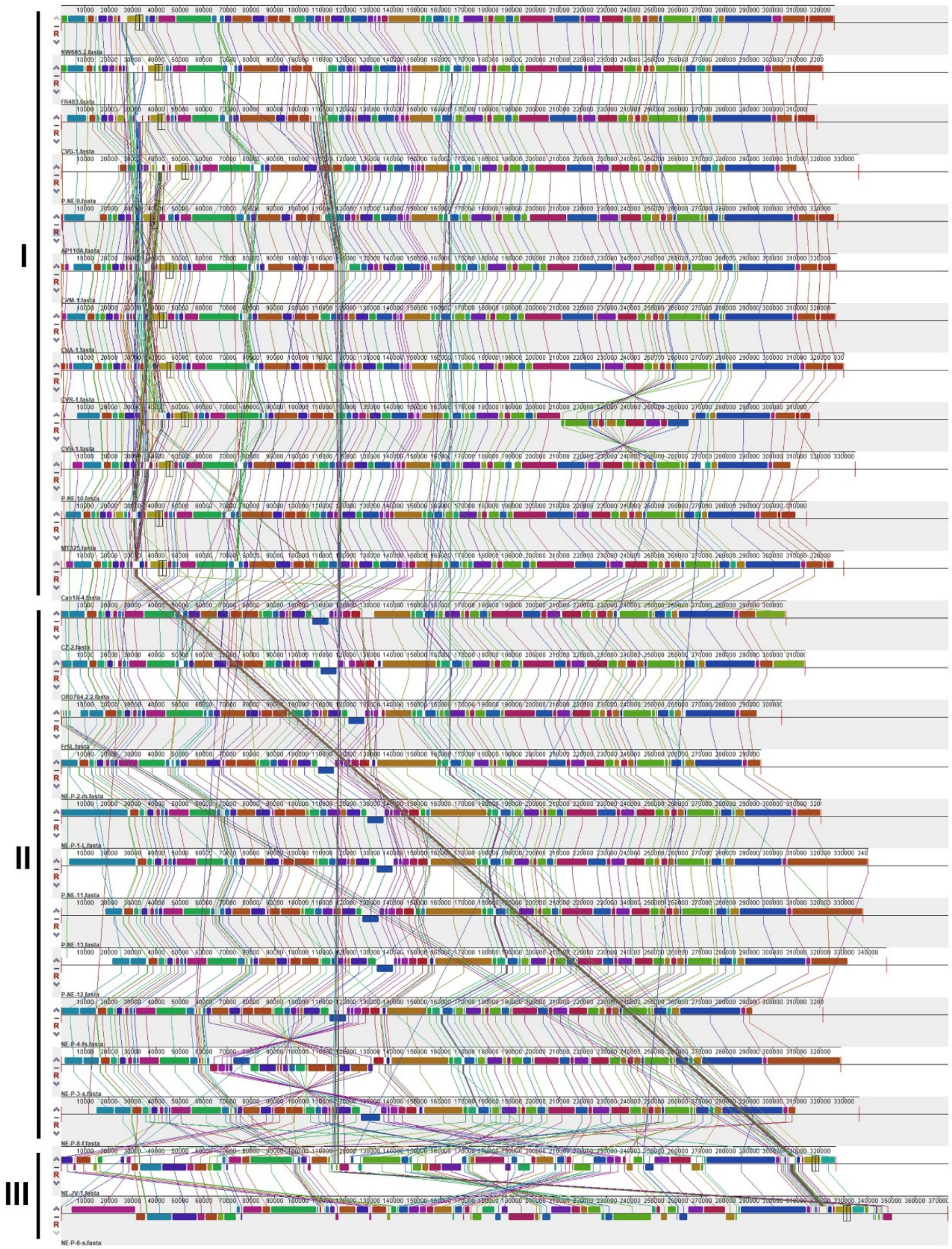

Figure S2: Synteny of all 25 betachloroviruses. A general good collinearity is observed among the isolates, except for the last two (species III), where inversions and translocations are observed compared to other isolates. Lines connect the collinear blocks. The three species are indicated on the right side of the panel with Roman numerals.

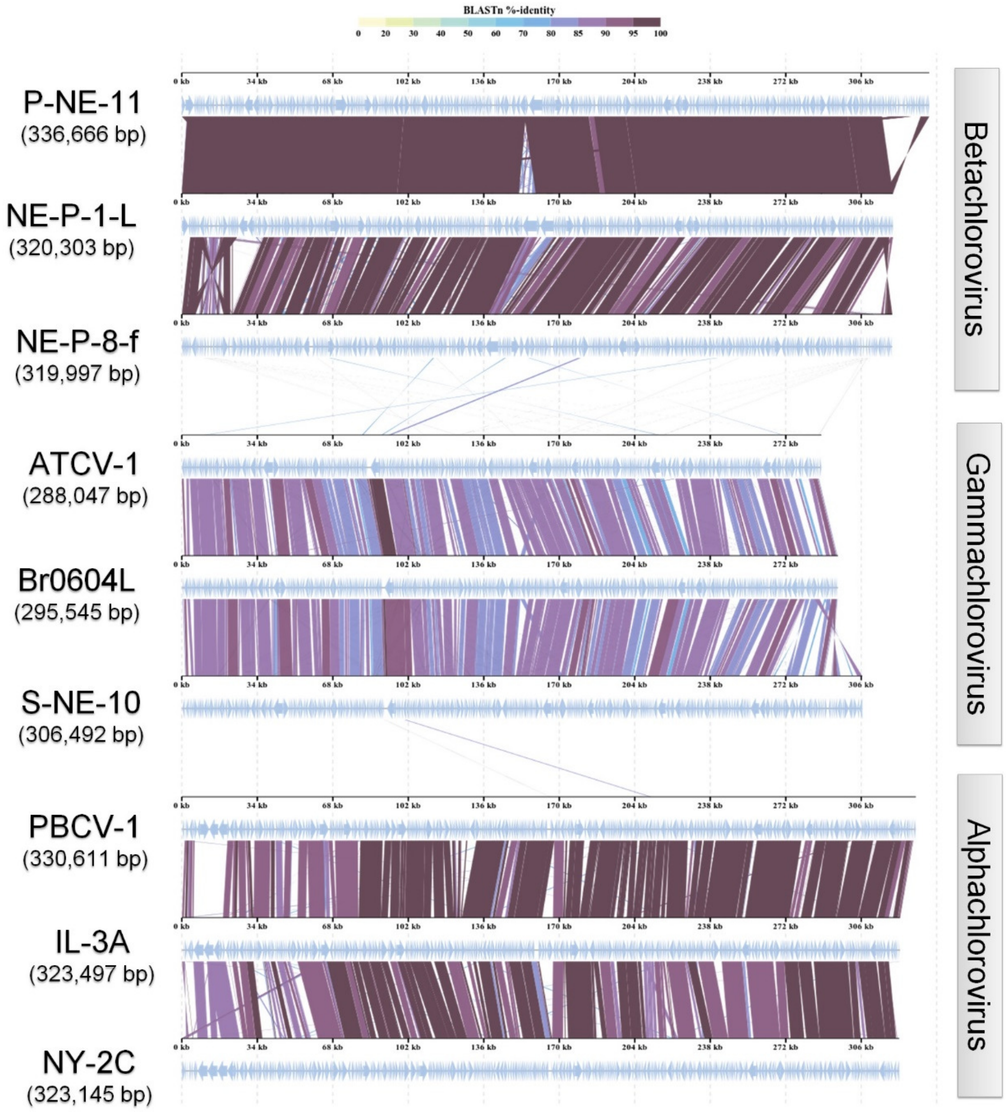

Figure S3: Comparative synteny among selected members of the 3 chlorovirus subgenera. A high degree of collinearity is observed among genomes within the same group, whereas comparisons between different groups reveal a markedly low level of synteny.

	NE-P-6-s	NE-JV-1	NE-P-8-f	NE-P-3-s	NE-P-4-fs	P-NE-12	P-NE-13	P-NE-11	NE-P-1-L	NE-P-2-m	Fr5L	OR0704.2.2	CZ-2	Can18-4	MT325	P-NE-10	CVB-1	CVR-1	CVA-1	CVM-1	AP110A	P-NE-9	CVG-1	FR483	NW665.2
NE-P-6-s	100,00	97,83	66,47	66,46	66,45	66,45	66,47	66,46	66,39	66,31	66,71	66,46	66,56	67,23	67,74	67,31	67,54	67,81	67,61	67,57	67,42	67,52	67,36	66,87	67,32
NE-JV-1	97,83	100,00	66,78	66,66	66,66	66,68	66,79	66,78	66,56	66,47	67,14	66,97	66,78	67,84	67,95	67,75	68,09	68,11	67,97	67,85	67,90	68,04	67,64	67,20	67,62
NE-P-8-f	66,47	66,78	100,00	99,75	99,62	99,77	96,20	95,85	96,10	96,25	95,91	95,17	95,34	91,28	91,88	91,50	91,80	91,88	91,68	92,14	91,22	91,98	92,11	91,09	91,80
NE-P-3-s	66,46	66,66	99,75	100,00	99,96	99,97	96,08	95,87	96,15	96,29	95,79	95,34	95,39	91,57	91,88	91,50	92,11	92,05	91,58	92,29	91,51	92,05	92,08	91,11	91,80
NE-P-4-fs	66,45	66,66	99,62	99,96	100,00	99,96	96,12	95,83	96,04	96,17	95,78	95,37	95,48	91,41	91,95	91,48	92,11	92,05	91,67	92,29	91,51	92,03	92,07	91,10	91,79
P-NE-12	66,45	66,68	99,77	99,97	99,96	100,00	96,11	95,88	96,16	96,30	95,80	95,35	95,28	91,41	91,89	91,52	91,95	92,05	91,57	92,13	91,35	92,05	92,07	91,10	91,79
P-NE-13	66,47	66,79	96,20	96,08	96,12	96,11	100,00	99,85	99,97	99,96	97,02	96,70	96,24	91,00	91,71	91,76	91,93	92,14	91,68	92,18	91,67	92,22	92,25	91,51	91,78
P-NE-11	66,46	66,78	95,85	95,87	95,83	95,88	99,85	100,00	99,82	99,70	96,86	96,72	96,46	90,95	91,64	91,66	91,90	92,21	91,72	92,13	91,71	92,18	92,14	91,52	91,69
NE-P-1-L	66,39	66,56	96,10	96,15	96,04	96,16	99,97	99,82	100,00	99,97	96,90	96,78	96,09	90,90	91,85	91,63	91,84	91,94	91,74	92,13	91,62	92,07	92,17	91,28	91,85
NE-P-2-m	66,31	66,47	96,25	96,29	96,17	96,30	99,96	99,70	99,97	100,00	96,97	96,58	96,10	91,13	91,95	91,76	91,76	91,90	91,73	92,20	91,74	92,27	92,35	91,31	91,81
Fr5L	66,71	67,14	95,91	95,79	95,78	95,80	97,02	96,86	96,90	96,97	100,00	96,02	96,29	91,41	92,42	92,08	92,31	92,27	92,32	92,51	92,20	92,54	91,95	91,84	91,87
OR0704.2.2	66,46	66,97	95,17	95,34	95,37	95,35	96,70	96,72	96,78	96,58	96,02	100,00	96,61	90,51	91,69	91,60	91,69	92,03	91,86	91,86	91,55	91,52	91,81	91,53	91,34
CZ-2	66,56	66,78	95,34	95,39	95,48	95,28	96,24	96,46	96,09	96,10	96,29	96,61	100,00	90,76	91,55	91,68	91,91	92,21	92,18	92,04	91,93	92,01	92,16	91,65	91,52
Can18-4	67,23	67,84	91,28	91,57	91,41	91,41	91,00	90,95	90,90	91,13	91,41	90,51	90,76	100,00	96,10	96,41	93,24	93,46	93,56	93,98	93,51	94,66	94,01	93,26	93,47
MT325	67,74	67,95	91,88	91,88	91,95	91,89	91,71	91,64	91,85	91,95	92,42	91,69	91,55	96,10	100,00	97,41	93,86	94,14	94,03	95,05	94,49	95,09	94,74	93,85	94,40
P-NE-10	67,31	67,75	91,50	91,50	91,48	91,52	91,76	91,66	91,63	91,76	92,08	91,60	91,68	96,41	97,41	100,00	94,18	94,27	94,08	95,29	93,96	95,70	94,76	93,62	94,04
CVB-1	67,54	68,09	91,80	92,11	92,11	91,95	91,93	91,90	91,84	91,76	92,31	91,69	91,91	93,24	93,86	94,18	100,00	94,97	94,95	95,17	94,62	95,06	94,76	94,55	94,04
CVR-1	67,81	68,11	91,88	92,05	92,05	92,05	92,14	92,21	91,94	91,90	92,27	92,03	92,21	93,46	94,14	94,27	94,97	100,00	99,91	96,53	96,23	95,40	95,33	94,66	94,44
CVA-1	67,61	67,97	91,68	91,58	91,67	91,57	91,68	91,72	91,74	91,73	92,32	91,86	92,18	93,56	94,03	94,08	94,95	99,91	100,00	96,52	95,99	95,31	95,29	94,47	94,43
CVM-1	67,57	67,85	92,14	92,29	92,29	92,13	92,18	92,13	92,13	92,20	92,51	91,86	92,04	93,98	95,05	95,29	95,17	96,53	96,52	100,00	96,27	95,79	95,48	94,64	95,12
AP110A	67,42	67,90	91,22	91,51	91,51	91,35	91,67	91,71	91,62	91,74	92,20	91,55	91,93	93,51	94,49	93,96	94,62	96,23	95,99	96,27	100,00	95,14	94,87	94,08	94,38
P-NE-9	67,52	68,04	91,98	92,05	92,03	92,05	92,22	92,18	92,07	92,27	92,54	91,52	92,01	94,66	95,09	95,60	95,06	95,40	95,31	95,79	95,14	100,00	97,11	95,18	95,12
CVG-1	67,36	67,64	92,11	92,08	92,07	92,07	92,25	92,14	92,17	92,35	91,95	91,81	92,16	94,01	94,74	94,76	94,76	95,33	95,29	95,48	94,87	97,11	100,00	94,54	94,80
FR483	66,87	67,20	91,09	91,11	91,10	91,10	91,51	91,52	91,28	91,31	91,84	91,53	91,65	93,26	93,85	93,62	94,55	94,66	94,47	94,64	94,08	95,18	94,54	100,00	94,74
NW665.2	67,32	67,62	91,80	91,80	91,79	91,79	91,78	91,69	91,85	91,81	91,87	91,34	91,52	93,47	94,40	94,04	94,04	94,44	94,43	95,12	94,38	95,12	94,80	94,74	100,00

Figure S4: Amino Acid Identity (AAI) analysis of betachloroviruses. Heatmap illustrating the protein-level similarity among betachloroviruses based on AAI values. Each square displays the corresponding percentage identity, with higher values shown in green and lower values in gray.

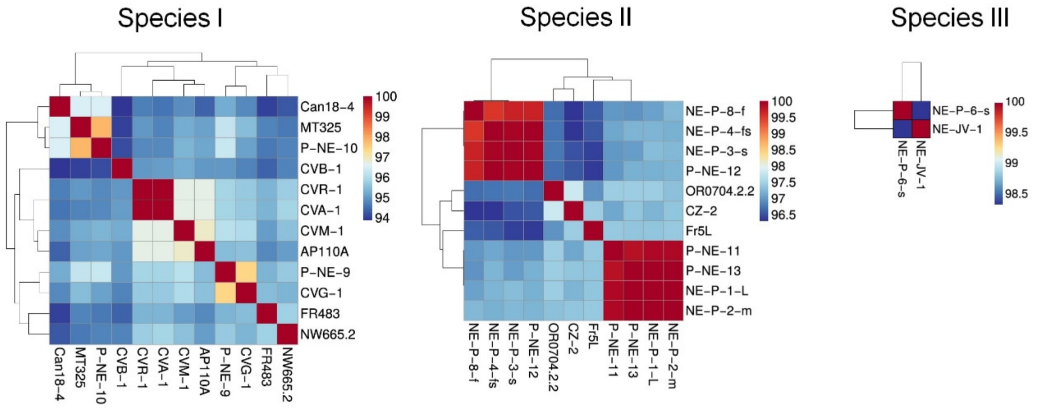

Figure S5: Average Nucleotide Identity of each betachlorovirus species. Clusters were defined using Pearson correlation.

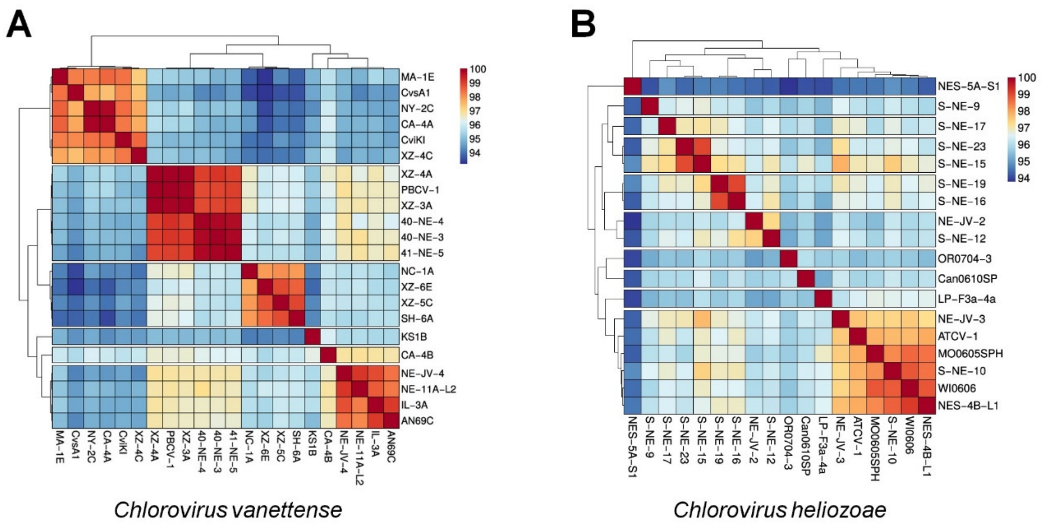

Figure S6: Average Nucleotide Identity of alphachlorovirus and gammachlorovirus. A) ANI with hierarchical clustering based on Pearson correlation for A) *Chlorovirus vanettense*; and B) *Chlorovirus heliozoae.*

Table S1: General genomic features and isolation information of betachloroviruses

Virus isolate	GC%	#CDS	#tRNA	Genome (Kbp)	Year of isolation	Continent	Country	GenBank accession
**AP110A**	44.3	374	9	327	NA	NA	NA	JX997154
**Can18-4**	44.7	383	10	329	1995	North America	Canada	JX997157
**CVA-1**	44.5	368	10	326	1984	Europe	Germany	JX997159
**CVB-1**	44.3	362	10	319	1984	Europe	Germany	JX997160
**CVG-1**	44.9	362	10	318	1984	Europe	Germany	JX997161
**CVM-1**	44.4	373	10	327	1984	Europe	Germany	JX997163
**CVR-1**	44.5	376	10	330	1984	Europe	Germany	JX997164
**CZ-2**	45.1	368	12	305	1995	Europe	Czech Republic	JX997166
**Fr5L**	44.7	385	12	303	1995	Europe	France	JX997167
**FR483**	44.6	365	8	321	1997	Europe	France	DQ890022
**MT325**	45.3	355	10	314	1996	North America	USA	DQ491001
**NE-JV-1**	46.7	378	5	326	2008	North America	USA	JX997176
**NW665.2**	44.1	373	9	325	1995	Europe	Norway	JX997181
**OR0704.2.2**	45	374	7	313	2007	North America	USA	JX997184
**NE-P-1-L**	45.3	364	12	320	2017	North America	USA	PV288763
**NE-P-2-m**	45.3	337	12	295	2017	North America	USA	PV288764
**NE-P-3-s**	45.1	385	9	328	2017	North America	USA	PV288765
**NE-P-4-fs**	44.9	385	9	321	2017	North America	USA	PV288766
**NE-P-6-s**	46.8	421	10	374	2017	North America	USA	PV288767
**NE-P-8-f**	44.6	401	8	336	2017	North America	USA	PV288768
**P-NE-9**	45.2	384	10	336	2017	North America	USA	PV288769
**P-NE-10**	45	379	9	334	2017	North America	USA	PV288770
**P-NE-11**	45.2	391	12	340	2020	North America	USA	PV288771
**P-NE-12**	45	400	9	348	2019	North America	USA	PV288772
**P-NE-13**	45.3	388	12	338	2018	North America	USA	PV288773

NA: Not available

Table S2: Average Nucleotide Identity values of betachloroviruses.

Query	Subject	ANI value
NE-P-1-L	NE-P-1-L	100
NE-P-1-L	P-NE-13	99.996
NE-P-1-L	NE-P-2-m	99.9903
NE-P-1-L	P-NE-11	99.9163
NE-P-1-L	OR0704.2.2	97.4241
NE-P-1-L	Fr5L	97.3548
NE-P-1-L	NE-P-3-s	97.2852
NE-P-1-L	CZ-2	97.2216
NE-P-1-L	NE-P-4-fs	97.2205
NE-P-1-L	P-NE-12	97.1734
NE-P-1-L	NE-P-8-f	97.0739
NE-P-1-L	P-NE-9	93.4216
NE-P-1-L	MT325	93.2207
NE-P-1-L	CVG-1	93.1288
NE-P-1-L	P-NE-10	93.1272
NE-P-1-L	CVM-1	92.9253
NE-P-1-L	CVR-1	92.8464
NE-P-1-L	CVB-1	92.8112
NE-P-1-L	CVA-1	92.8053
NE-P-1-L	AP110A	92.6307
NE-P-1-L	FR483	92.6038
NE-P-1-L	NW665.2	92.4828
NE-P-1-L	Can18-4	92.4519
NE-P-1-L	NE-P-6-s	83.5877
NE-P-1-L	NE-JV-1	83.2172
NE-P-2-m	NE-P-2-m	100
NE-P-2-m	P-NE-13	99.9957
NE-P-2-m	NE-P-1-L	99.9911
NE-P-2-m	P-NE-11	99.9098
NE-P-2-m	Fr5L	97.3971
NE-P-2-m	CZ-2	97.3553
NE-P-2-m	OR0704.2.2	97.3423
NE-P-2-m	NE-P-8-f	97.2515
NE-P-2-m	NE-P-3-s	97.2421
NE-P-2-m	P-NE-12	97.2146
NE-P-2-m	NE-P-4-fs	97.1895
NE-P-2-m	P-NE-9	93.2958
NE-P-2-m	CVG-1	93.2523
NE-P-2-m	MT325	93.237
NE-P-2-m	CVR-1	93.1594
NE-P-2-m	CVA-1	93.1302
NE-P-2-m	P-NE-10	92.9961
NE-P-2-m	CVB-1	92.8336
NE-P-2-m	FR483	92.7469
NE-P-2-m	CVM-1	92.6946
NE-P-2-m	AP110A	92.6269
NE-P-2-m	NW665.2	92.5777
NE-P-2-m	Can18-4	92.4572

NE-P-2-m	NE-JV-1	84.2366
NE-P-2-m	NE-P-6-s	83.8786
NE-P-3-s	NE-P-3-s	100
NE-P-3-s	P-NE-12	99.9482
NE-P-3-s	NE-P-4-fs	99.942
NE-P-3-s	NE-P-8-f	99.6813
NE-P-3-s	NE-P-2-m	97.2121
NE-P-3-s	NE-P-1-L	97.1948
NE-P-3-s	P-NE-11	97.1179
NE-P-3-s	P-NE-13	97.0562
NE-P-3-s	OR0704.2.2	96.6485
NE-P-3-s	CZ-2	96.506
NE-P-3-s	Fr5L	96.2885
NE-P-3-s	P-NE-9	93.3273
NE-P-3-s	CVG-1	93.2192
NE-P-3-s	MT325	93.126
NE-P-3-s	CVM-1	93.0089
NE-P-3-s	AP110A	92.7097
NE-P-3-s	CVB-1	92.6691
NE-P-3-s	P-NE-10	92.6497
NE-P-3-s	Can18-4	92.6395
NE-P-3-s	NW665.2	92.5955
NE-P-3-s	FR483	92.5829
NE-P-3-s	CVR-1	92.5622
NE-P-3-s	CVA-1	92.4953
NE-P-3-s	NE-JV-1	84.6082
NE-P-3-s	NE-P-6-s	84.4385
NE-P-4-fs	NE-P-4-fs	100
NE-P-4-fs	P-NE-12	99.9734
NE-P-4-fs	NE-P-3-s	99.9542
NE-P-4-fs	NE-P-8-f	99.5574
NE-P-4-fs	NE-P-1-L	97.3037
NE-P-4-fs	P-NE-11	97.2189
NE-P-4-fs	P-NE-13	97.192
NE-P-4-fs	NE-P-2-m	97.0735
NE-P-4-fs	OR0704.2.2	96.611
NE-P-4-fs	CZ-2	96.396
NE-P-4-fs	Fr5L	96.3782
NE-P-4-fs	CVG-1	93.1815
NE-P-4-fs	P-NE-9	93.1733
NE-P-4-fs	MT325	93.1551
NE-P-4-fs	CVM-1	93.1317
NE-P-4-fs	P-NE-10	92.9717
NE-P-4-fs	CVB-1	92.8958
NE-P-4-fs	NW665.2	92.8921
NE-P-4-fs	FR483	92.8483
NE-P-4-fs	CVA-1	92.8259
NE-P-4-fs	AP110A	92.8177
NE-P-4-fs	CVR-1	92.815
NE-P-4-fs	Can18-4	92.6445
NE-P-4-fs	NE-JV-1	84.5462
NE-P-4-fs	NE-P-6-s	83.8613
AP110A	AP110A	100

AP110A	CVM-1	96.8803
AP110A	CVR-1	96.7868
AP110A	CVA-1	96.7452
AP110A	CVG-1	95.6335
AP110A	P-NE-9	95.3918
AP110A	MT325	95.2662
AP110A	NW665.2	95.0424
AP110A	P-NE-10	95.0165
AP110A	CVB-1	94.8428
AP110A	FR483	94.8031
AP110A	Can18-4	94.4064
AP110A	NE-P-4-fs	92.6537
AP110A	Fr5L	92.61
AP110A	P-NE-13	92.5601
AP110A	P-NE-11	92.5219
AP110A	NE-P-2-m	92.4975
AP110A	NE-P-8-f	92.4825
AP110A	CZ-2	92.4732
AP110A	P-NE-12	92.4557
AP110A	OR0704.2.2	92.3869
AP110A	NE-P-3-s	92.3106
AP110A	NE-P-1-L	92.1987
AP110A	NE-P-6-s	85.3097
AP110A	NE-JV-1	85.0112
Can18-4	Can18-4	100
Can18-4	P-NE-10	96.6504
Can18-4	MT325	96.2439
Can18-4	P-NE-9	95.2109
Can18-4	CVG-1	94.8193
Can18-4	CVM-1	94.7785
Can18-4	AP110A	94.5105
Can18-4	CVA-1	94.3737
Can18-4	CVR-1	94.3451
Can18-4	NW665.2	94.2863
Can18-4	FR483	93.8269
Can18-4	CVB-1	93.4053
Can18-4	P-NE-11	92.5028
Can18-4	NE-P-1-L	92.4337
Can18-4	NE-P-2-m	92.4159
Can18-4	P-NE-13	92.371
Can18-4	CZ-2	92.3428
Can18-4	Fr5L	92.317
Can18-4	P-NE-12	92.1486
Can18-4	NE-P-8-f	92.1123
Can18-4	NE-P-4-fs	92.1114
Can18-4	NE-P-3-s	91.9562
Can18-4	OR0704.2.2	91.9324
Can18-4	NE-P-6-s	86.8097
Can18-4	NE-JV-1	86.7082
CVA-1	CVA-1	100
CVA-1	CVR-1	99.9603
CVA-1	CVM-1	96.95
CVA-1	AP110A	96.7593

CVA-1	CVG-1	95.876
CVA-1	NW665.2	95.8578
CVA-1	P-NE-9	95.8268
CVA-1	FR483	95.4019
CVA-1	CVB-1	95.1427
CVA-1	P-NE-10	95.034
CVA-1	MT325	94.9398
CVA-1	Can18-4	94.5577
CVA-1	CZ-2	93.1111
CVA-1	P-NE-11	93.0319
CVA-1	NE-P-4-fs	92.9666
CVA-1	NE-P-8-f	92.913
CVA-1	NE-P-2-m	92.901
CVA-1	Fr5L	92.8888
CVA-1	P-NE-13	92.8514
CVA-1	OR0704.2.2	92.8157
CVA-1	NE-P-3-s	92.8079
CVA-1	P-NE-12	92.7366
CVA-1	NE-P-1-L	92.7104
CVA-1	NE-JV-1	84.7927
CVA-1	NE-P-6-s	84.7451
CVB-1	CVB-1	100
CVB-1	CVM-1	95.5481
CVB-1	P-NE-9	95.2273
CVB-1	CVR-1	95.095
CVB-1	CVA-1	95.081
CVB-1	CVG-1	95.0353
CVB-1	AP110A	94.9999
CVB-1	NW665.2	94.7616
CVB-1	FR483	94.377
CVB-1	P-NE-10	94.3359
CVB-1	MT325	94.1952
CVB-1	Can18-4	93.8756
CVB-1	Fr5L	93.1094
CVB-1	NE-P-4-fs	92.9403
CVB-1	NE-P-3-s	92.9345
CVB-1	NE-P-8-f	92.893
CVB-1	OR0704.2.2	92.7925
CVB-1	CZ-2	92.7644
CVB-1	P-NE-11	92.7313
CVB-1	NE-P-1-L	92.7246
CVB-1	P-NE-13	92.6802
CVB-1	P-NE-12	92.6591
CVB-1	NE-P-2-m	92.5966
CVB-1	NE-P-6-s	87.0751
CVB-1	NE-JV-1	86.1932
CVG-1	CVG-1	100
CVG-1	P-NE-9	96.9909
CVG-1	CVM-1	95.9968
CVG-1	CVR-1	95.7095
CVG-1	MT325	95.6929
CVG-1	CVA-1	95.6683
CVG-1	AP110A	95.5612

CVG-1	FR483	95.4702
CVG-1	NW665.2	95.3741
CVG-1	P-NE-10	95.1436
CVG-1	Can18-4	94.8561
CVG-1	CVB-1	94.7459
CVG-1	CZ-2	93.3714
CVG-1	P-NE-11	93.3648
CVG-1	P-NE-13	93.2217
CVG-1	Fr5L	93.1002
CVG-1	NE-P-2-m	93.0269
CVG-1	NE-P-1-L	92.9315
CVG-1	OR0704.2.2	92.9072
CVG-1	NE-P-4-fs	92.896
CVG-1	NE-P-3-s	92.8744
CVG-1	P-NE-12	92.8723
CVG-1	NE-P-8-f	92.6755
CVG-1	NE-P-6-s	86.4637
CVG-1	NE-JV-1	85.8733
CVM-1	CVM-1	100
CVM-1	AP110A	96.9339
CVM-1	CVA-1	96.7147
CVM-1	CVR-1	96.7132
CVM-1	P-NE-9	95.9728
CVM-1	CVG-1	95.7761
CVM-1	NW665.2	95.5411
CVM-1	CVB-1	95.2696
CVM-1	MT325	95.2087
CVM-1	P-NE-10	95.0654
CVM-1	FR483	95.0454
CVM-1	Can18-4	94.8796
CVM-1	CZ-2	93.2526
CVM-1	NE-P-1-L	93.0247
CVM-1	P-NE-11	92.9525
CVM-1	P-NE-13	92.9313
CVM-1	P-NE-12	92.918
CVM-1	Fr5L	92.9164
CVM-1	NE-P-8-f	92.8314
CVM-1	NE-P-4-fs	92.8086
CVM-1	OR0704.2.2	92.7959
CVM-1	NE-P-3-s	92.7153
CVM-1	NE-P-2-m	92.6965
CVM-1	NE-P-6-s	86.2346
CVM-1	NE-JV-1	85.6787
CVR-1	CVR-1	99.9996
CVR-1	CVA-1	99.9574
CVR-1	CVM-1	96.9206
CVR-1	AP110A	96.7195
CVR-1	P-NE-9	95.9817
CVR-1	CVG-1	95.8494
CVR-1	NW665.2	95.7516
CVR-1	FR483	95.406
CVR-1	CVB-1	95.017
CVR-1	P-NE-10	94.9663

CVR-1	MT325	94.8988
CVR-1	Can18-4	94.7135
CVR-1	CZ-2	93.1771
CVR-1	Fr5L	93.0973
CVR-1	NE-P-4-fs	92.9909
CVR-1	NE-P-8-f	92.9708
CVR-1	P-NE-11	92.9375
CVR-1	NE-P-2-m	92.8427
CVR-1	OR0704.2.2	92.8175
CVR-1	NE-P-3-s	92.772
CVR-1	P-NE-13	92.7659
CVR-1	P-NE-12	92.7256
CVR-1	NE-P-1-L	92.5114
CVR-1	NE-JV-1	84.9598
CVR-1	NE-P-6-s	84.9076
CZ-2	CZ-2	100
CZ-2	OR0704.2.2	97.5615
CZ-2	NE-P-2-m	97.3464
CZ-2	P-NE-13	97.2933
CZ-2	NE-P-1-L	97.2745
CZ-2	P-NE-11	97.2388
CZ-2	Fr5L	97.1963
CZ-2	P-NE-12	96.5482
CZ-2	NE-P-3-s	96.4869
CZ-2	NE-P-4-fs	96.3274
CZ-2	NE-P-8-f	96.3152
CZ-2	P-NE-9	93.6508
CZ-2	CVG-1	93.4991
CZ-2	MT325	93.3714
CZ-2	NW665.2	93.1381
CZ-2	CVM-1	93.1375
CZ-2	CVA-1	93.0879
CZ-2	CVR-1	93.0801
CZ-2	P-NE-10	93.0718
CZ-2	AP110A	93.0373
CZ-2	FR483	92.9091
CZ-2	CVB-1	92.7673
CZ-2	Can18-4	92.5777
CZ-2	NE-JV-1	84.5086
CZ-2	NE-P-6-s	83.3139
Fr5L	Fr5L	100
Fr5L	P-NE-13	97.4697
Fr5L	CZ-2	97.436
Fr5L	NE-P-1-L	97.3733
Fr5L	P-NE-11	97.3696
Fr5L	NE-P-2-m	97.2466
Fr5L	OR0704.2.2	97.0823
Fr5L	NE-P-8-f	96.5796
Fr5L	NE-P-4-fs	96.5402
Fr5L	NE-P-3-s	96.4132
Fr5L	P-NE-12	96.3699
Fr5L	CVG-1	93.0872
Fr5L	P-NE-9	93.0322

Fr5L	CVA-1	92.9896
Fr5L	CVR-1	92.9679
Fr5L	MT325	92.9488
Fr5L	CVB-1	92.9269
Fr5L	P-NE-10	92.8906
Fr5L	CVM-1	92.8045
Fr5L	FR483	92.5975
Fr5L	AP110A	92.5532
Fr5L	Can18-4	92.5067
Fr5L	NW665.2	92.4012
Fr5L	NE-JV-1	86.0643
Fr5L	NE-P-6-s	85.2311
FR483	FR483	100
FR483	NW665.2	95.7916
FR483	CVG-1	95.6651
FR483	CVA-1	95.5438
FR483	CVR-1	95.4854
FR483	P-NE-9	95.4152
FR483	CVM-1	95.4144
FR483	AP110A	95.176
FR483	P-NE-10	94.3943
FR483	MT325	94.3738
FR483	CVB-1	94.2579
FR483	Can18-4	94.0551
FR483	CZ-2	92.8488
FR483	NE-P-3-s	92.8307
FR483	NE-P-4-fs	92.7799
FR483	NE-P-8-f	92.7458
FR483	NE-P-2-m	92.7123
FR483	P-NE-11	92.6389
FR483	P-NE-12	92.6361
FR483	NE-P-1-L	92.5464
FR483	P-NE-13	92.4887
FR483	OR0704.2.2	92.4282
FR483	Fr5L	92.3995
FR483	NE-JV-1	85.9592
FR483	NE-P-6-s	85.4545
MT325	MT325	100
MT325	P-NE-10	97.8316
MT325	Can18-4	96.5523
MT325	P-NE-9	96.3099
MT325	CVG-1	95.483
MT325	CVM-1	95.2633
MT325	AP110A	95.1362
MT325	CVR-1	95.0103
MT325	CVA-1	94.7718
MT325	FR483	94.7399
MT325	NW665.2	94.624
MT325	CVB-1	94.0492
MT325	NE-P-2-m	93.3861
MT325	P-NE-13	93.3594
MT325	CZ-2	93.335
MT325	P-NE-11	93.2608

MT325	NE-P-1-L	93.2556
MT325	NE-P-3-s	93.1861
MT325	NE-P-4-fs	93.1264
MT325	NE-P-8-f	93.0665
MT325	Fr5L	93.0339
MT325	OR0704.2.2	92.9229
MT325	P-NE-12	92.8796
MT325	NE-JV-1	86.8549
MT325	NE-P-6-s	86.0518
NE-JV-1	NE-JV-1	99.9999
NE-JV-1	NE-P-6-s	98.2504
NE-JV-1	P-NE-9	87.7035
NE-JV-1	P-NE-10	87.6953
NE-JV-1	MT325	87.5851
NE-JV-1	Fr5L	87.191
NE-JV-1	Can18-4	86.504
NE-JV-1	NW665.2	86.4807
NE-JV-1	FR483	86.2952
NE-JV-1	CVB-1	86.2255
NE-JV-1	CVG-1	85.9864
NE-JV-1	AP110A	85.7322
NE-JV-1	CVA-1	85.4036
NE-JV-1	CVR-1	85.3587
NE-JV-1	CZ-2	85.1607
NE-JV-1	CVM-1	85.1237
NE-JV-1	OR0704.2.2	84.9366
NE-JV-1	NE-P-8-f	84.6336
NE-JV-1	P-NE-11	84.5805
NE-JV-1	NE-P-4-fs	84.4999
NE-JV-1	NE-P-3-s	84.4959
NE-JV-1	P-NE-13	84.1886
NE-JV-1	P-NE-12	84.1045
NE-JV-1	NE-P-1-L	84.0758
NE-JV-1	NE-P-2-m	83.6441
NW665.2	NW665.2	100
NW665.2	FR483	95.82
NW665.2	P-NE-9	95.7362
NW665.2	CVR-1	95.6608
NW665.2	CVA-1	95.6209
NW665.2	CVM-1	95.5582
NW665.2	CVG-1	95.4943
NW665.2	AP110A	95.439
NW665.2	MT325	94.835
NW665.2	CVB-1	94.5044
NW665.2	P-NE-10	94.3686
NW665.2	Can18-4	94.2934
NW665.2	CZ-2	93.1506
NW665.2	OR0704.2.2	92.9994
NW665.2	P-NE-13	92.9603
NW665.2	P-NE-11	92.8649
NW665.2	NE-P-1-L	92.8614
NW665.2	NE-P-2-m	92.8237
NW665.2	NE-P-3-s	92.7325

NW665.2	P-NE-12	92.6717
NW665.2	NE-P-8-f	92.5889
NW665.2	Fr5L	92.4726
NW665.2	NE-P-4-fs	92.4657
NW665.2	NE-JV-1	86.8217
NW665.2	NE-P-6-s	86.5662
OR0704.2.2	OR0704.2.2	100
OR0704.2.2	CZ-2	97.8279
OR0704.2.2	P-NE-11	97.5091
OR0704.2.2	P-NE-13	97.457
OR0704.2.2	NE-P-1-L	97.4102
OR0704.2.2	NE-P-2-m	97.2184
OR0704.2.2	Fr5L	96.967
OR0704.2.2	NE-P-3-s	96.7816
OR0704.2.2	NE-P-4-fs	96.7539
OR0704.2.2	P-NE-12	96.7117
OR0704.2.2	NE-P-8-f	96.6822
OR0704.2.2	CVR-1	93.2023
OR0704.2.2	CVA-1	93.1436
OR0704.2.2	MT325	93.1349
OR0704.2.2	CVG-1	92.9853
OR0704.2.2	P-NE-9	92.9218
OR0704.2.2	CVB-1	92.8963
OR0704.2.2	P-NE-10	92.7208
OR0704.2.2	CVM-1	92.5906
OR0704.2.2	NW665.2	92.5266
OR0704.2.2	FR483	92.4954
OR0704.2.2	AP110A	92.4895
OR0704.2.2	Can18-4	92.3375
OR0704.2.2	NE-JV-1	84.4387
OR0704.2.2	NE-P-6-s	82.9736
P-NE-10	P-NE-10	100
P-NE-10	MT325	97.9941
P-NE-10	Can18-4	96.5656
P-NE-10	P-NE-9	96.3559
P-NE-10	CVG-1	95.4212
P-NE-10	CVM-1	95.366
P-NE-10	AP110A	95.2007
P-NE-10	CVA-1	94.8599
P-NE-10	CVR-1	94.8578
P-NE-10	NW665.2	94.78
P-NE-10	FR483	94.6473
P-NE-10	CVB-1	94.1208
P-NE-10	NE-P-4-fs	93.2801
P-NE-10	CZ-2	93.2379
P-NE-10	P-NE-12	93.2265
P-NE-10	P-NE-11	93.1251
P-NE-10	NE-P-3-s	93.0916
P-NE-10	NE-P-1-L	93.0729
P-NE-10	NE-P-8-f	92.9003
P-NE-10	NE-P-2-m	92.7851
P-NE-10	P-NE-13	92.7625
P-NE-10	Fr5L	92.738

P-NE-10	OR0704.2.2	92.5774
P-NE-10	NE-P-6-s	87.0412
P-NE-10	NE-JV-1	85.8049
P-NE-11	P-NE-11	100
P-NE-11	P-NE-13	99.8992
P-NE-11	NE-P-1-L	99.8972
P-NE-11	NE-P-2-m	99.8887
P-NE-11	OR0704.2.2	97.4404
P-NE-11	Fr5L	97.4144
P-NE-11	NE-P-4-fs	97.1225
P-NE-11	CZ-2	97.1056
P-NE-11	P-NE-12	97.0153
P-NE-11	NE-P-8-f	97.0087
P-NE-11	NE-P-3-s	96.9981
P-NE-11	P-NE-10	93.3501
P-NE-11	CVG-1	93.2554
P-NE-11	P-NE-9	93.1516
P-NE-11	MT325	93.0648
P-NE-11	CVR-1	92.7947
P-NE-11	CVM-1	92.7756
P-NE-11	CVA-1	92.7729
P-NE-11	CVB-1	92.7663
P-NE-11	NW665.2	92.7424
P-NE-11	FR483	92.5611
P-NE-11	AP110A	92.434
P-NE-11	Can18-4	92.4014
P-NE-11	NE-JV-1	83.6711
P-NE-11	NE-P-6-s	83.0787
P-NE-12	P-NE-12	99.9999
P-NE-12	NE-P-3-s	99.9938
P-NE-12	NE-P-4-fs	99.9445
P-NE-12	NE-P-8-f	99.6861
P-NE-12	P-NE-13	97.2301
P-NE-12	NE-P-1-L	97.2294
P-NE-12	P-NE-11	97.1411
P-NE-12	NE-P-2-m	97.0924
P-NE-12	OR0704.2.2	96.8441
P-NE-12	CZ-2	96.4827
P-NE-12	Fr5L	96.3601
P-NE-12	P-NE-9	93.3935
P-NE-12	MT325	93.063
P-NE-12	CVM-1	92.9923
P-NE-12	CVG-1	92.9638
P-NE-12	CVR-1	92.9501
P-NE-12	AP110A	92.9207
P-NE-12	CVA-1	92.8365
P-NE-12	FR483	92.8187
P-NE-12	P-NE-10	92.7851
P-NE-12	Can18-4	92.7611
P-NE-12	CVB-1	92.7026
P-NE-12	NW665.2	92.6403
P-NE-12	NE-JV-1	84.354
P-NE-12	NE-P-6-s	83.7793

P-NE-13	P-NE-13	100
P-NE-13	NE-P-1-L	99.9969
P-NE-13	NE-P-2-m	99.991
P-NE-13	P-NE-11	99.8504
P-NE-13	OR0704.2.2	97.431
P-NE-13	Fr5L	97.3723
P-NE-13	CZ-2	97.3528
P-NE-13	NE-P-4-fs	97.2355
P-NE-13	P-NE-12	97.1928
P-NE-13	NE-P-3-s	97.0437
P-NE-13	NE-P-8-f	96.9659
P-NE-13	P-NE-9	93.623
P-NE-13	MT325	93.4266
P-NE-13	CVG-1	93.3704
P-NE-13	CVA-1	93.258
P-NE-13	CVR-1	93.2271
P-NE-13	CVB-1	93.1181
P-NE-13	P-NE-10	93.1112
P-NE-13	CVM-1	93.0853
P-NE-13	NW665.2	93.0714
P-NE-13	Can18-4	92.8298
P-NE-13	AP110A	92.4945
P-NE-13	FR483	92.4925
P-NE-13	NE-JV-1	84.3587
P-NE-13	NE-P-6-s	83.4207
NE-P-6-s	NE-P-6-s	100
NE-P-6-s	NE-JV-1	98.3546
NE-P-6-s	P-NE-10	88.0564
NE-P-6-s	P-NE-9	87.439
NE-P-6-s	NW665.2	87.3879
NE-P-6-s	MT325	87.328
NE-P-6-s	Can18-4	86.7509
NE-P-6-s	CVG-1	86.7468
NE-P-6-s	FR483	86.3255
NE-P-6-s	Fr5L	86.3136
NE-P-6-s	CVB-1	86.1627
NE-P-6-s	CVR-1	86.1126
NE-P-6-s	CVA-1	86.1041
NE-P-6-s	AP110A	86.0983
NE-P-6-s	CVM-1	85.648
NE-P-6-s	CZ-2	84.4393
NE-P-6-s	NE-P-3-s	84.3697
NE-P-6-s	P-NE-12	84.1607
NE-P-6-s	NE-P-4-fs	84.1091
NE-P-6-s	P-NE-13	84.0095
NE-P-6-s	NE-P-8-f	83.8943
NE-P-6-s	NE-P-1-L	83.8451
NE-P-6-s	OR0704.2.2	83.6118
NE-P-6-s	P-NE-11	83.4864
NE-P-6-s	NE-P-2-m	83.4559
NE-P-8-f	NE-P-8-f	100
NE-P-8-f	NE-P-3-s	99.6899
NE-P-8-f	P-NE-12	99.6517

NE-P-8-f	NE-P-4-fs	99.5236
NE-P-8-f	NE-P-1-L	97.0628
NE-P-8-f	P-NE-13	97.0104
NE-P-8-f	P-NE-11	96.9726
NE-P-8-f	NE-P-2-m	96.7628
NE-P-8-f	OR0704.2.2	96.7184
NE-P-8-f	CZ-2	96.4159
NE-P-8-f	Fr5L	96.3095
NE-P-8-f	P-NE-9	93.3316
NE-P-8-f	CVG-1	93.2721
NE-P-8-f	MT325	93.2471
NE-P-8-f	CVM-1	93.2243
NE-P-8-f	CVR-1	93.1836
NE-P-8-f	CVA-1	93.1062
NE-P-8-f	P-NE-10	93.0985
NE-P-8-f	FR483	92.8777
NE-P-8-f	CVB-1	92.8738
NE-P-8-f	NW665.2	92.8179
NE-P-8-f	AP110A	92.7335
NE-P-8-f	Can18-4	92.5225
NE-P-8-f	NE-JV-1	85.0123
NE-P-8-f	NE-P-6-s	84.7887
P-NE-9	P-NE-9	100
P-NE-9	CVG-1	97.4044
P-NE-9	P-NE-10	96.2787
P-NE-9	MT325	96.2073
P-NE-9	CVM-1	96.1161
P-NE-9	CVA-1	95.8698
P-NE-9	CVR-1	95.8458
P-NE-9	NW665.2	95.6918
P-NE-9	AP110A	95.5185
P-NE-9	FR483	95.4004
P-NE-9	Can18-4	95.2638
P-NE-9	CVB-1	95.0357
P-NE-9	P-NE-13	93.4618
P-NE-9	CZ-2	93.4386
P-NE-9	NE-P-1-L	93.4255
P-NE-9	P-NE-11	93.3393
P-NE-9	NE-P-3-s	93.2556
P-NE-9	OR0704.2.2	93.195
P-NE-9	NE-P-4-fs	93.1848
P-NE-9	NE-P-2-m	93.1039
P-NE-9	P-NE-12	93.0834
P-NE-9	Fr5L	93.0655
P-NE-9	NE-P-8-f	93.0647
P-NE-9	NE-P-6-s	86.9266
P-NE-9	NE-JV-1	86.6672
